# A trial to determine whether septic shock-reversal is quicker in pediatric patients randomized to an early goal-directed fluid-sparing strategy versus usual care (SQUEEZE): study protocol for a pilot randomized controlled trial

**DOI:** 10.1186/s13063-016-1689-2

**Published:** 2016-11-22

**Authors:** Melissa J. Parker, Lehana Thabane, Alison Fox-Robichaud, Patricia Liaw, Karen Choong, For the Canadian Critical Care Trials Group and the Canadian Critical Care Translational Biology Group

**Affiliations:** 1 Division of Pediatric Critical Care, Department of Pediatrics, McMaster Children’s Hospital and McMaster University, HSC 3E-20,1280 Main Street West, Hamilton, ON L8S 4K1 Canada; 2 Department of Clinical Epidemiology and Biostatistics, McMaster University, 1200 Main Street West, Hamilton, ON L8N 3Z5 Canada; 3 Division of Emergency Medicine, Department of Pediatrics, the Hospital for Sick Children, University of Toronto, 555 University Avenue, Toronto, ON M5G 1X8 Canada; 4 Department of Anesthesia, McMaster University, 1200 Main Street West, Hamilton, ON L8N 3Z5 Canada; 5 Biostatistics Unit,/FSORC, St Joseph’s Healthcare Hamilton, 3rd floor Martha Wing, 50 Charlton Avenue East, Hamilton, ON L8N 4A6 Canada; 6 Department of Medicine, McMaster University, DBRI, Rm C5–106 and 107, 237 Barton Street East, Hamilton, ON L8L 2X2 Canada

**Keywords:** Fluid therapy, Resuscitation, Shock, Sepsis, Pediatrics

## Abstract

**Background:**

Current pediatric septic shock resuscitation guidelines from the American College of Critical Care Medicine focus on the early and goal-directed administration of intravascular fluid followed by vasoactive medication infusions for persistent and fluid-refractory shock. However, accumulating adult and pediatric data suggest that excessive fluid administration is associated with worse patient outcomes and even increased risk of death. The optimal amount of intravascular fluid required in early pediatric septic shock resuscitation prior to the initiation of vasoactive support remains unanswered.

**Methods/design:**

The SQUEEZE Pilot Trial is a pragmatic, two-arm, parallel-group, open-label, prospective pilot randomized controlled trial. Participants are children aged 29 days to under 18 years with suspected or confirmed septic shock and a need for ongoing resuscitation. Eligible participants are enrolled under an exception to consent process and randomly assigned via concealed allocation to either the Usual Care (control) or Fluid Sparing (intervention) resuscitation strategy. The primary objective of this pilot trial is to determine feasibility, based on the ability to enroll participants and to adhere to the study protocol. The primary outcome measure by which success will be determined is participant enrollment rate ("pass" defined as at least two participants/site/month, recognizing that enrollment may be slower during the run-in phase). Secondary objectives include assessing (1) appropriateness of eligibility criteria, and (2) completeness of clinical outcomes to inform the endpoints for the planned multisite trial. To support the nested translational study, SQUEEZE-D, we will also evaluate the feasibility of describing cell-free DNA (a procoagulant molecule with prognostic utility) in blood samples obtained from children enrolled into the SQUEEZE Pilot Trial at baseline and at 24 h.

**Discussion:**

The optimal degree of fluid resuscitation and the timing of initiation of vasoactive support in order to achieve recommended therapeutic targets in children with septic shock remains unanswered. No prospective study to date has examined this important question for children in developed countries including Canada. Recruitment for the SQUEEZE Pilot Trial opened on 6 January 2014. Findings will inform the feasibility of the planned multicenter trial to answer our overall research question.

**Trial registration:**

ClinicalTrials.gov Identifier NCT01973907, registered on 23 October 2013.

**Electronic supplementary material:**

The online version of this article (doi:10.1186/s13063-016-1689-2) contains supplementary material, which is available to authorized users.

## Background

### Rationale

Septic shock remains one of the most significant and potentially preventable causes of death in children worldwide, with pediatric mortality rates ranging from 15 to 70% [1, 2]. Current pediatric Surviving Sepsis Guidelines [3, 4] from the American College of Critical Care Medicine (ACCM) emphasize an early and goal-directed approach to resuscitation [5–7]. These guidelines suggest that fluid resuscitation should be aggressive with repeated intravenously (IV) administered fluid boluses of 20 mL/kg, such that some children may require as much as 200 mL/kg of fluid to achieve therapeutic endpoints [2]. The guidelines also recommend initiation of vasoactive agents at the stage of “fluid-refractory shock,” i.e., when there is persistent hypoperfusion despite at least 60 mL/kg IV fluid [3]. While evidence suggests that adhering to the resuscitation goals and guidelines of the ACCM may improve mortality and functional morbidity [8, 9], fluid resuscitation guidelines from the ACCM were derived primarily from observational studies and expert opinion [3].

Aggressive fluid administration in septic shock has recently been called into question [10]. Accumulating adult [11–14] and pediatric data [15–18] suggest that excessive fluid resuscitation in patients with septic shock is associated with increased morbidity and mortality. This has sparked a furious debate in both the adult and pediatric medical literature on how “aggressive” fluid resuscitation should be, given that the main morbidity and mortality in septic shock is due not to refractory hypotension, but to end-organ failure due in part to massive fluid overload [19]. The overall objective of our research program is, therefore, to evaluate whether a fluid-sparing strategy, that involves earlier initiation and preferential escalation of vasoactive medications to achieve ACCM goal-directed targets, results in improved clinical outcomes for children experiencing septic shock.

### Relevant medical literature

Mortality in pediatric septic shock has significantly improved since the introduction of rapid fluid resuscitation in the first “golden” hour of resuscitation [6–9, 20]. Subsequent improvements in pediatric septic shock survival have been attributed to adherence to the first iteration of the ACCM septic shock guidelines, and the use of goal-directed targets [21, 22]. However, the largest and most publicized pediatric trial of fluid resuscitation in children with suspected septic shock (FEAST trial), published in the *New England Journal of Medicine* in 2011, demonstrated an increased mortality among children treated with aggressive fluid resuscitation in comparison to the conservative fluid resuscitation arm [15]. These results sparked a flurry of commentaries and attempts to explain these unexpected findings [23–25]. The FEAST trial was conducted in sub-Saharan Africa, and enrolled a significant proportion of children with malaria. As a result, the pediatric critical care community clearly acknowledges that these results, while important, are not necessarily generalizable to developed countries such as Canada. These results did, however, fuel further discussion and debate regarding the optimum fluid resuscitation in the course of goal-directed therapy in septic shock.

Emerging publications in the Intensive Care Unit (ICU) medical literature suggest that excessive, compared to conservative, fluid administration in adults with septic shock worsens outcomes such as duration of mechanical ventilation [17, 26], complications related to the third-spacing of fluids [27, 28], length of ICU stay [17, 26], and mortality [11–14]. Recent systematic reviews reveal a paucity of randomized controlled trial (RCT) evidence, other than the FEAST trial, examining the impact of fluid resuscitation on mortality in children with septic shock [29, 30]. This raises the important question of whether children in developed countries would also benefit from fluid-sparing resuscitation. A goal-directed fluid-sparing strategy would, by default, require earlier initiation and preferential escalation of vasoactive medications to target ACCM hemodynamic goals [3]. There are potential adverse effects attributable to the earlier initiation of vasoactive medications that may outweigh those resulting from an aggressive fluid administration strategy, providing further justification for this study [27, 28, 31–33]. The optimal degree of fluid resuscitation and the timing of initiation of vasoactive support in order to achieve therapeutic targets in children with septic shock remain unanswered. No prospective study to date has examined this important question for children in developed countries including Canada.

#### Overall research question

In pediatric patients with septic shock, does a fluid-sparing strategy to achieve ACCM therapeutic goals result in improved clinical outcomes without an increased risk of adverse events compared to the usual care of aggressive fluid resuscitation, as currently recommended by the ACCM guidelines?

#### Pilot trial primary research question

Is it feasible to conduct a multicentre trial using this protocol?

### Explanation for choice of comparators

There is currently not clinical equipoise to randomize children with septic shock to a “No bolus” intervention. For this reason, the only sensible way to test emerging concepts while producing high-quality evidence is to investigate comparators of a “fluid-sparing” strategy versus “usual care,” where the latter is fluid liberal.

## Methods/design

The SQUEEZE Pilot Trial is a pragmatic two-arm, parallel-group, open-label, prospective pilot RCT. We will use this pilot RCT to determine the feasibility and inform the appropriate methodological design of the larger definitive trial [34, 35].

The primary objective of the SQUEEZE Pilot Trial is to determine the feasibility of a large multicentre RCT to answer our overall research question. Secondary objectives are to assess the (1) appropriateness of the eligibility criteria, and (2) completeness of clinical outcomes of interest for the main study to inform the design of the full-scale trial. Clinical outcomes under consideration for the definitive trial include clinical endpoints related to:

1. Clinical course and procedures, e.g., Pediatric Intensive Care Unit (PICU) admission rate, Length of Stay, measures of organ dysfunction, i.e., PELOD-2 score, ventilator-free days, mortality, invasive lines and procedures, laboratory and microbiological findings
2. Short-term hemodynamic outcomes, e.g., time to shock-reversal, cardiovascular indices
3. Adverse events: (i) complications potentially attributable to fluid overload, e.g., pulmonary edema, pleural effusion requiring drainage, abdominal compartment syndrome, and (ii) complications potentially attributable to inotrope/vasopressor use, e.g., signs of digital soft tissue ischemia, need for revision amputation

The pilot trial primary and secondary study objectives will be evaluated according to the outcomes listed in Table 1. We will also collect additional data related to study process, resource, and management aspects of feasibility to inform conduct of the definitive multicentre RCT. The proposed primary objective for the definitive SQUEEZE Trial is to determine whether time to shock-reversal is quicker in pediatric patients with septic shock treated with a Fluid Sparing resuscitation strategy versus Usual Care.

**Table 1:** Summary of Pilot Trial outcomes

Pilot Trial outcomes	Analysis	Pass threshold
SQUEEZE		
Primary outcomes		
1. Participant enrollment rate^a^ Consent rate for continued participation Missed eligible patients	Simple proportion Simple proportion Simple proportion	≥2/month(/site) Not applicable Not applicable
2. Protocol adherence: ability to initiate study procedures within 1 h of randomization	Simple proportion	Not applicable
Secondary outcomes		
1. Appropriateness of eligibility criteria as evidenced by the ability to identify and enroll participants in a timely manner	Descriptive	Not applicable
2. Completeness of the clinical outcomes of interest to inform the design of the future multicenter trial	Descriptive	Not applicable
3. We will also assess considerations related to study process, resource, and management aspects of feasibility	Descriptive	Not applicable
SQUEEZE-D		
Primary outcome		
1. The proportion of SQUEEZE participants for whom cell-free deoxyribonucleic acid (cfDNA) can be described	Simple proportion	Not applicable
Secondary outcomes		
1. The availability of the required samples from patients enrolled into SQUEEZE and	Simple proportion	Not applicable
2. We will also assess considerations related to study process, resource, and management aspects of feasibility which impact upon the ability to process and test samples to inform the design of a larger-scale study	Descriptive	Not applicable

^a^Recognizing that enrollment may be slower during the initial run-in phase

The SQUEEZE Pilot Trial includes a nested translational study, SQUEEZE-D. The overall objective of SQUEEZE-D is to describe the levels of plasma cell-free deoxyribonucleic acid (cfDNA) in pediatric septic shock. cfDNA is released by activated neutrophils and is a potent trigger of blood coagulation. In adult patients with severe sepsis, cfDNA has high discriminative power to predict ICU mortality [36]. The primary objective of SQUEEZE-D in the context of the pilot trial is to evaluate the feasibility of describing cfDNA in blood samples obtained for clinical purposes at baseline and 24 h in children enrolled in the SQUEEZE Pilot Trial. The secondary objectives of SQUEEZE-D are to determine (1) the availability of the required samples from patients enrolled in SQUEEZE and (2) the process, resource, and management aspects of feasibility which impact upon the ability to process and test samples to inform the design of a larger-scale study. The proposed primary objective of SQUEEZE-D in the definitive trial is to determine the predictive value of cfDNA on length of time in shock.

**Fig. 1:**
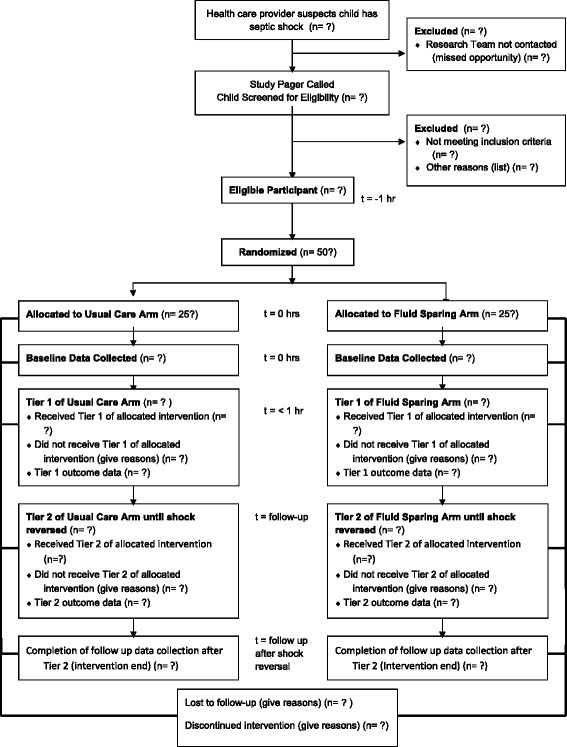
Flow of participants

The detailed pilot trial protocol is organized in accordance with the Standard Protocol Items: Recommendations for Interventional Trials (SPIRIT) guidelines, with items corresponding to the SPIRIT 2013 checklist (Additional file 1) [37, 38]. Required items, such as the World Health Organization Trial Registration Data Set (Additional file 2), a schedule of enrollment, interventions, and assessments (Additional file 3), and a participant flow diagram (Fig. 1) are included here. The protocol was registered at ClinicalTrials.gov (NCT01973907) prior to enrollment of the first participant.

### Setting

The SQUEEZE Pilot Trial currently includes McMaster Children’s Hospital (Hamilton, Canada); however, we will consider adding external Canadian site(s) once the trial is established. Participating sites will be listed on the ClinicalTrials.gov trial registration page.

### Participants

Patients presenting to the emergency department, or admitted to an inpatient ward (including the PICU) at participating sites who meet the following eligibility criteria:

***Inclusion criteria:***

Inclusion criteria for 1 and 3 must be answered *yes* to be eligible.

1. Age 29 days to under 18 years of age
2. Persistent signs of shock defined as one or more of the following:
3. Vasoactive medication dependence (need for vasoactive drug for hemodynamic support)
4. Hypotension (systolic blood pressure (SBP) and/or mean blood pressure (MBP) below the 5th percentile for age)
5. Abnormal perfusion, defined as the presence of two or more of the following:
  abnormal capillary refill (CR) (CR <1 s (flash) or CR ≥3 s (delayed)), tachycardia (heart rate (HR) above the 95th percentile for age)
  decreased level of consciousness
  decreased urine output
6. Suspected or confirmed septic shock
7. Fluid resuscitation threshold met. Patient has received within the previous 6 h a minimum of:
8. 40 mL/kg of isotonic crystalloid (0.9% normal saline or Ringer’s lactate), and/or colloid (5% albumin) as fluid bolus therapy administered IV for participants <50 kg
*or*
9. ^δ^2 L of isotonic crystalloid (0.9% normal saline or Ringer’s lactate), and/or colloid (5% albumin) as IV fluid bolus therapy for participants ≥50 kg

3. Fluid-refractory septic shock as defined by the presence of 2a, 2b, and 2c.
  *Adapted from the International Pediatric Sepsis Consensus Conference: definitions for sepsis and organ dysfunction in pediatrics [1]
^δ^Based on the adult Surviving Sepsis Guidelines initial targets for fluid resuscitation [4]

***Exclusion criteria:***

i) Patient admitted to the Neonatal Intensive Care Unit (NICU)
ii) Full active resuscitative treatment is not within the goals of care
iii) Shock secondary to causes other than sepsis (i.e., obvious signs of cardiogenic shock, anaphylactic shock, hemorrhagic shock, spinal shock)
iv) Patients requiring resuscitation in the operating room or Post-anesthetic Care Unit
v) Previous enrollment in this trial, where known by the research team

### Interventions

Patients will be randomized in a 1:1 ratio to either: (1) Usual Care, or (2) Fluid Sparing.

For all participants, care providers will be provided with a copy of the hemodynamic goals as specified in the ACCM Surviving Sepsis Guidelines and instructed that they should escalate treatment to achieve these targets according to the assigned intervention. A copy of the current ACCM Guidelines will also be provided to promote adherence to best practices in both study arms for aspects of patient care not impacted by the study intervention.

An illustration of the two study arms in ACCM Guideline format is provided in Additional file 4, while the ACCM Goal Directed Targets are summarized in Additional file 5. Table 2 provides a detailed description of the two-tiered study intervention. Each intervention tier provides direction regarding the use of bolus fluid therapy and vasoactive medications. A one-page flow diagram will be used in the clinical setting to provide simple directions regarding implementation of the intervention.

**Table 2:** Detailed description of the SQUEEZE study arms

Intervention tier	Usual Care arm	Fluid Sparing arm
Tier 1	Usual Care	Early initiation of vasoactive medications to spare fluid
Bolus fluid therapy^a,b^	• Following randomization, further isotonic fluid bolus therapy [crystalloid (0.9% normal saline or Ringer’s lactate) or colloid (5% albumin)] may be administered in *any volume* and as requested by the caring physician	• Following randomization, further isotonic fluid bolus therapy [crystalloid (0.9% normal saline or Ringer’s lactate) or colloid (5% albumin)] *should be avoided* and provided only if required due to: 1. delay in the ability to immediately initiate vasoactive medication(s) and/or 2. to treat intravascular hypovolemia. The reason/indication for administration of further fluid bolus therapy prior to the initiation of vasoactive medications must be documented
Vasoactive medication^c^	• The decision to initiate vasoactive medication(s) is at the discretion of the treating physician. Vasoactive support should not be started until the participant has received a *minimum of 60 mL/kg (3 L for participants ≥50 kg)* of isotonic fluid as boluses (includes fluid boluses received in the 6 h prior to randomization) • The choice of initial vasoactive medication and the initial dose is to be at the discretion of the caring physician	• Vasoactive medication(s) should be initiated immediately following randomization • The choice of initial vasoactive medication and the initial dose is to be at the discretion of the caring physician
Tier 2	Usual Care	Preferential escalation of vasoactive medications
Bolus fluid therapy^a,b^	• Further isotonic fluid bolus therapy may be administered at the discretion of the caring physician • The *type and dose* of any further isotonic fluid bolus therapy is at the discretion of the caring physician	• Further isotonic fluid bolus therapy may be administered by the caring physician *to treat documented inadequate intravascular filling/preload* • If further isotonic fluid bolus therapy is provided, the *dose* provided should be in 5–10-mL/kg aliquots (*250-500 mL for participants ≥50 kg*) with the lowest acceptable volume preferred and the indication for administration documented • Aliquots of isotonic fluid bolus therapy may be administered “back-to-back” if required to address inadequate intravascular volume status • The *type* of isotonic fluid bolus therapy provided is at the discretion of the caring physician
Vasoactive medication^c^	• If initiated, vasoactive medication(s) may be titrated (increased, decreased, or discontinued) at the discretion of the caring physician • Additional vasoactive medication(s) may be initiated at the discretion of the caring physician	• *Escalation of vasoactive medications should be the first line to achieve hemodynamic goals* (provided intravascular volume status is judged to be adequate) • The initiated vasoactive medication(s) may be titrated (increased, decreased, or discontinued) at the discretion of the caring physician • Additional vasoactive medication(s) may be initiated at the discretion of the caring physician
Intervention end	• When the patient is free from vasoactive medication support and shock is reversed *or* the patient is placed on mechanical circulatory support,e.g., extracorporeal membrane oxygenation (ECMO) *or* death occurs	• When the patient is free from vasoactive medication support and shock is reversed *or* the patient is placed on mechanical circulatory support, e.g., ECMO *or* death occurs

^a^Bolus: a (fluid) bolus is a discrete volume of fluid prescribed to be administered intravascularly (intravenous (IV) or intraosseous (IO)) over a defined period of time (ranging from stat, i.e., as fast as possible to *typically* no greater than 60 min). A fluid bolus *typically* ranges in size from usually not less than 5 mL/kg (250 mL for participants ≥50 kg) to 20 mL/kg (1 L for participants ≥50 kg, although some clinicians may use per kilogram dosing in larger patients). A documented medical order is required for a fluid bolus. Routine fluid replacement is not considered to be bolus(es)

^b^Fluid therapy: isotonic crystalloid or colloid solutions which include 0.9% normal saline, Ringer’s lactate, and 5% albumin

^c^Vasoactive medications are administered by intravascular (IV or IO) infusion and include: dobutamine, dopamine, epinephrine, norepinephrine, vasopressin, phenylephrine, milrinone

Criteria for discontinuing or modifying allocated interventions for a given trial participant:

We will allow for exit criteria from the study protocol as follows:

i) Participant or their Substitute Decision Maker (SDM) withdraws consent for ongoing study participation
ii) Change in the medical goals of care for a study participant, e.g., decision to limit escalation of resuscitative therapies and/or withdrawal of life-sustaining supportive measures
iii) Confirmatory evidence that the participant is suffering from another form of shock other than septic shock, e.g., occult hemorrhage
iv) The Most Responsible Physician (MRP) believes that ongoing patient management according to the assigned intervention will lead to patient harm

The site principal investigator (PI) or their delegate should be contacted if any of these situations arise to discuss the specific circumstances. If the PI withdraws a participant from the study, clear and objective reason(s) should be recorded.

### Strategies to improve adherence to intervention protocols

For study participants, we will post an alert on the front of the medical chart and inside their room, e.g., at the head of the bed advising of trial enrollment and the assigned intervention. In the Fluid Sparing arm, a fluid bolus record requires the medical team to provide justification for any fluid boluses administered to the participant. Data will be reviewed in light of the assigned intervention and any protocol deviations will be documented. Study process feasibility includes protocol adherence [35]. We will document and report the following protocol deviations: failure to (1) implement study procedures within 1 h of randomization, (2) prescribe fluid boluses in accordance with protocol, (3) prescribe vasoactive medications in accordance with protocol, and (4) provide notification of enrollment to the participant or SDM per protocol. The importance of protocol adherence will be routinely reinforced. There will be no restrictions with respect to concomitant care and interventions.

### Study outcomes

Pilot trial outcomes are summarized in Table 1. The proposed primary outcome for the full study is *time to shock-reversal*. We have defined time to shock-reversal as the amount of time (in hours) from allocation until shock is reversed. *Shock-reversal* is defined based on the ACCM therapeutic targets, when all of the following criteria have been met:

1. Free from all vasoactive medication support
2. Normalization of HR (above the 5th, and below the 95th percentile for age)
3. Normalization of blood pressure (SBP and MBP above the 5th percentile for age)
4. CR <3 s

Shock-reversal criteria will be assessed as documented in the medical record. Shock will not be determined to be reversed unless the patient has been free from vasoactive infusions for 24 h. If a participant is placed on mechanical circulatory support, such as extracorporeal membrane oxygenation (ECMO), or if death occurs during the intervention phase, shock will be coded as “never reversed.”

### Measurement of cfDNA (SQUEEZE-D)

The citrated plasma tube (CPT) used for determination of the International Normalized Ratio (INR) and partial thromboplastin time (PTT) is the same collection tube used for cfDNA. We plan to use the CPT tube as the source of plasma for cfDNA determination. The cfDNA levels will be measured at baseline and at 24 h as previously described in the laboratory of Drs. Fox-Robichaud and Liaw at the Thrombosis and Atherosclerosis Research Institute [36].

### Sample size

The target sample size for the SQUEEZE pilot, based on the study objective of evaluating feasibility, is 50 subjects (25 patients per arm). This is consistent with current guidelines for sample size justification for pilot trials [39]. We will consider adjusting the pilot trial sample size upward to provide an opportunity for the new site(s) to recruit sufficient subjects to allow for assessment of external feasibility.

### Screening and recruitment

Patients will be screened for eligibility from the following patient care areas: emergency department, medical and surgical wards, and the Pediatric Critical Care Unit (PCCU). Posters and information sessions will be used to promote the study to physician and nursing staff, and trainees. The research coordinator and/or PI will be paged for any potentially eligible patient, e.g., suspected sepsis, receiving a fluid bolus. If/when a patient is screened and determined to meet study eligibility criteria, and provided there is no objection by the MRP or their delegate, the randomization procedures will be executed immediately and the patient enrolled. This trial will use an exception to consent (deferred consent) process (see “Ethical Considerations”).

### Allocation sequence generation, allocation concealment, participant enrollment, and communication of participant assignment

The allocation sequence will be created by the Biostatistics Unit and this information will be kept secret from, and inaccessible by, the investigators. The allocation sequence will be computer-generated and utilize simple randomization, with no stratification or blocking. The allocation sequence will be implemented through a third party computer-based process accessible via telephone on a 24-h basis. A research assistant or one of the site investigators will enroll eligible participants into the study and be responsible for communicating the assigned intervention.

### Blinding

The investigators, research staff, and treating health care providers will all be blinded to the allocation sequence. It will not be possible to blind the investigators, treating physicians, or bedside nursing staff from participant assignment as these individuals will need to be aware of, and implement, the intervention. We will evaluate the feasibility of blinding the research assistants (who will obtain Ultrasonic Cardiac Output Monitor (USCOM™) measurements) to participant treatment assignment. The data analysts will be blinded to treatment assignment through use of a numeric code in the trial database.

### Activities to promote participant retention and follow-up

We do not anticipate difficulties with participant follow-up given that those enrolled will be receiving close monitoring and management for suspected septic shock. Data collected prior to approaching participants/SDMs for written consent to continue study participation will be retained for all participants enrolled in the trial. Where a protocol deviation occurs, this information including the reason will be noted and data collection will otherwise continue according to protocol.

### Data collection methods

Participant demographic data and SQUEEZE outcome data will be collected from the hospital chart by a research assistant or one of the investigators, trained in use of the Data Collection Forms. The investigators and research assistants will be trained in the use of the noninvasive USCOM™ ultrasound device for study purposes at sites where this technology is available. At the times specified within the study protocol, and where feasible, USCOM™ measurements will be obtained by one of these individuals and recorded on the related Data Collection Form. Dr. Fox-Robichaud will be responsible for oversight of SQUEEZE-D outcome data collection.

### Data collection, management, and security

Trial data will be collected by trained research staff or one of the study investigators according to study procedures. Data will be recorded on a paper-based Data Collection Form and then entered into the electronic REDCap Case Report Form (CRF) by trained research staff [40]. Paper files will be kept locked and secure in accordance with local laws and regulations. SQUEEZE-D outcome data will be stored in the Team Sepsis database, which is located at the Thrombosis and Atherosclerosis Research Institute (TaARI).

### Statistical methods for analyzing primary and secondary outcomes

Baseline characteristics and outcome variables (both primary and secondary) will be summarized using descriptive summary measures. Data analysis will consist of calculating simple proportions for the feasibility outcomes. Analysis of feasibility outcomes will be descriptive expressed as an estimate with 95% confidence interval. Clinical outcomes (blinded) will be estimated for the purposes of estimating the sample size calculations for the planned definitive trial. Analysis and reporting of the results with follow the Consolidated Standards of Reporting Trials (CONSORT) guidelines for reporting RCTs [41, 42]. Because we plan to include (roll-in) the pilot trial participants in the definitive trial, we do not plan to perform analyses of clinical outcomes. All analyses will be performed using SAS 9.2 (Cary, NC, USA).

### Data Safety and Monitoring Board

Normally a Data Safety and Monitoring Board (DSMB) is required for studies involving vulnerable populations including children. However, we decided not to set up a DSMB for this pilot trial given the minimal risk attributable to participation. Further, the study duration is too short for the DSMB process [43].

### Steering Committee

The Steering Committee will consist of Drs. Parker, Choong, Thabane, and Fox-Robichaud and will oversee the execution and conduct of the study. The PI and the Steering Committee will monitor adverse events (AEs), and report all serious adverse events (SAEs) to the Research Ethics Board (REB).

### Interim analyses, stopping rules, auditing

As this is a pilot trial, there will be no interim analysis and no predetermined stopping rules. There are no planned audits. Our study may be subjected to audit by the REB(s) of participating sites. As a clinical trial, our study may also be subjected to audit by Health Canada.

### Harms

The number and type of SAEs that occur will be monitored by the trial Steering Committee. There are many published complications that can normally occur as a result of septic shock and/or its treatment including multiorgan dysfunction and death. The vast majority of children in North America now survive septic shock; they may fully recover or they may be left with residual/permanent disability. The decision as to whether an AE is a SAE will be at the discretion of the PI based on proposed guiding principles for academic critical care research [44]. All SAEs along with the Steering Committee’s interpretation of attribution, will be reported to the REB. Data regarding SAEs will be collected and reported.

### Research Ethics Board approval and communication of protocol modifications

Approval to conduct this pilot trial has been granted by the Hamilton Integrated Research Ethics Board (Project # 13-295). Each participating site must seek and receive full REB approval for trial participation prior to enrolling any participants.

Any modifications to trial procedures must be communicated to, reviewed by, and approved by the Hamilton Integrated REB, as well as the REBs of any other participating sites. The PI will be responsible for communicating approved changes in the trial protocol to the research coordinator.

### Consent and assent procedures

Pediatric septic shock is a recognized medical emergency and, given that our study will evaluate a time-sensitive resuscitation protocol, we will use an exception to consent process (deferred consent) to achieve timely enrollment, randomization, and initiation of study procedures. The use of an exception to consent process for research evaluating treatment of emergency conditions has precedent [45–47] and is supported in Canada by the Tri-Council Policy Statement: Ethical Conduct for Research Involving Humans (TCPS2) (Chapter 3, Article 3.8) [48]. The use of an exception to consent process to conduct this study is ethical because some research cannot be conducted without the use of alternate consent models [48].

The participant and/or the SDM for the participant (for children who are incapable of consent) will be notified that they have been enrolled into a trial. At the earliest appropriate opportunity, the research coordinator or one of the study investigators will approach the participant or SDM to provide detailed information about the study and seek consent for ongoing participation. It will be made clear that ongoing study participation is voluntary and that any decision regarding further participation will not influence the medical care provided. Participants who are incapable of providing consent will be approached for assent as applicable. We will also monitor participants on an ongoing basis for signs of dissent.

### Plans for collection and use of personal health information

Collection of participant identifiers will be limited to those determined to be necessary for trial purposes. Participants will be assigned a unique identifying code number, with participant identifiers kept separate from other trial data collected. The file linking participant code numbers to identifying information will be maintained on a password-protected computer while paper CRFs will be kept locked within a filing cabinet in a secure area.

### Endpoint adjudication

We will adjudicate the time of resolution of septic shock, according to our study definitions, and post-randomization exclusions, e.g., patients for whom it is subsequently determined that shock was clearly due to another cause.

### Data Management Team

The Data Management Team includes the investigators, the research coordinator, and the REDCap super administrator. The REDCap super administrator controls the setup of projects and, together with the PI, establishes user accounts and user rights for the research team.

### Authorship eligibility

Criteria for authorship on any manuscript disseminating study results will be determined in accordance with the statement from the International Committee of Medical Journal Editors [49]. Medical/professional writers will not be involved in manuscript preparation.

### Plans for communication of trial results and data access

Plans for communication of trial results include presentation at one or more national or international scientific meetings and publication in a peer-reviewed journal. The investigators plan to eventually make the final trial data set publicly available.

## Discussion

We describe a pragmatic pilot RCT evaluating a fluid-sparing intervention in pediatric septic shock. Fluid resuscitation is a hot topic in need of rigorous study to inform optimal patient care. Our research aims to fill this gap with high-quality evidence. Resuscitation trials face unique challenges, given that interventions are frequently time-sensitive in vulnerable patients. This is certainly true of our study and supports the need for an initial pilot trial to evaluate feasibility prior to proceeding with a larger study. Pilot trials also offer an opportunity to test and refine trial processes and procedures before embarking on a more costly and complex endeavor.

Making our trial pragmatic was a priority to best reflect how the intervention would work in day-to-day practice. For this reason the trial contains very few exclusion criteria and, apart from these, aims to enroll all children with persistent signs of shock in the setting of suspected or proven infection. We recognize and accept that “usual care” for some study participants, such as those with premorbid cardiac conditions or renal impairment, may approach that of the intervention strategy. However, randomization should lead to these and other patients with analogous circumstances being evenly distributed between the study arms in any large-scale trial. Also pragmatic, we suggest that clinicians follow the ACCM guidelines for treatments apart from those impacted by the study intervention; however, we do not rigidly require guideline adherence. Some elements of the Surviving Sepsis Guidelines are currently under debate as a result of recent high-profile publications while others are in the process of being studied. Of particular note, the PROCESS, ARISE, and PROMISE trials challenge (in adults) the utility of aggressive treatment to meet ACCM goals such as central venous pressure (CVP) and hemoglobin targets [50–52]. However, we do not consider these recent publications a threat to our investigation. Rather, evidence supporting a move away from aggressively targeting measures, such as CVP, actually supports adherence to our fluid-sparing intervention. Changing attitudes towards other targets, such as transfusion thresholds, should also be evenly distributed between groups as is expected for both known and unknown confounders.

A number of considerations went into defining the study intervention. Without doubt a large volume of fluid may be administered in the initial stages of septic shock resuscitation prior to the initiation of vasoactive support. Early initiation of vasoactive support was the only acceptable way to mitigate this, with permissive hypotension the alternative. In children, hypotension is a late clinical finding and we expect that inaction in the face of hypotension would be unacceptable to any clinicians providing care for children with septic shock (including our team). An important caveat, however, is that physiologically adequate end-organ perfusion and age-based blood pressure targets are not necessarily one in the same. Nonetheless, the ACCM currently defines goal-directed hemodynamic targets by age – at least during the early stages of resuscitation (Additional file 5) [3, 4]. While the early initiation of vasoactive medications could define the intervention alone, we felt that this was likely insufficient to result in meaningful fluid sparing among any participants but those with the mildest cases of septic shock. To be effective, a fluid-sparing intervention likely requires an ongoing strategy to limit fluid administration after vasoactive support has been initiated.

We also sought to define our control arm pragmatically while being mindful of the need to achieve between-group separation. Consistent with the ACCM guidelines, we request that vasoactive support not be initiated in participants randomized to the Usual Care arm until at least 60 mL/kg (3 L for patients >50 kg) of isotonic fluid boluses has been administered from the time of shock onset. We expect between-group separation to be potentially challenging to evaluate, given the small pilot trial sample size and resulting increased risk of imbalance in potentially important confounders such as illness severity. Depending on the nature of these findings, further modifications to the study inclusion criteria and/or the intervention may be required.

The planned primary outcome for the definitive trial is time to shock-reversal. We consider time to shock-reversal a clinically meaningful outcome since this measure is objective and impacts the need for critical care resources. While we will measure and report PICU Length of Stay, this outcome may be influenced by a host of factors apart from the patient’s condition, rendering it less useful. Another candidate primary outcome is organ dysfunction, as measured by PELOD-2 [53]. While we will report mortality data, mortality is not an appropriate or feasible primary outcome for many pediatric trials due to high survivorship in children compared to adults for many conditions and a comparatively smaller number of available participants.

We expect that one of the most contentious aspects of this study will be our use of an exception to consent process. Considering the rapid rate at which septic shock resuscitation unfolds, as well as the life-threatening emergency nature of the situation, it is impracticable to seek prospective informed consent and any attempt to do so would risk coercion. Early enrollment in the course of resuscitation is also required from a scientific perspective to achieve a meaningful impact of the intervention. We are genuinely interested in SDM experiences with the exception to consent process and research is also underway to evaluate this specifically [54].

The optimal degree of fluid resuscitation and the timing of initiation of vasoactive support in order to achieve therapeutic targets in children with septic shock remain unanswered. In their systematic review Ford et al. concluded “The most important direction for future research is the applicability of the findings of the FEAST trial to other populations and settings” [16]. SQUEEZE will help to answer this very question in children with access to advanced critical care in countries such as Canada. The pilot trial described herein represents the first step in achieving this aim.

### Trial status

Active recruitment at the time of manuscript submission. Recruitment is now closed.

## Additional files

Additional file 1: Title of data: SPIRIT 2013 checklist. Description of data: completed SPIRIT 2013 checklist: recommended items to address in a clinical trial protocol and related documents. (PDF 108 kb)
Additional file 2: Title of data: items from the World Health Organization Trial Registration Data Set. Description of data: items from the World Health Organization Trial Registration Data Set. (PDF 110 kb)
Additional file 3: Title of data: schedule of enrollment, interventions, and assessments. Description of data: schedule of enrollment, interventions, and assessments. (PDF 155 kb)
Additional file 4: Title of data: SQUEEZE study algorithm as Illustrated in ACCM Guideline Format. Description of data: SQUEEZE study algorithm as Illustrated in ACCM Guideline Format. (PDF 1162 kb)
Additional file 5: Title of data: ACCM Goal Directed Targets from the Surviving Sepsis Guidelines. Description of data: summary of ACCM Goal Directed Targets from the Surviving Sepsis Guidelines [3, 4]. (PDF 78 kb)

## Abbreviations

ACCM: American College of Critical Care Medicine
AE: Adverse event
CBS: Canadian Blood Services
CIHR: Canadian Institutes of Health Research
CPT: Citrated plasma tube
CR: Capillary refill
CRF: Case Report Form
DSMB: Data Safety and Monitoring Board
ECMO: Extracorporeal membrane oxygenation
HHS: Hamilton Health Sciences
HIREB: Hamilton Integrated Research Ethics Board
HR: Heart rate
ICU: Intensive Care Unit
INR: International Normalized Ratio
IO: Intraosseous
IV: Intravascular
MBP: Mean blood pressure
MCH: McMaster Children’s Hospital
MRP: Most Responsible Physician
PCCU: Pediatric Critical Care Unit
PI: Principal investigator
PICU: Pediatric Intensive Care Unit
PTT: Partial thromboplastin time
RCT: Randomized controlled trial
REB: Research Ethics Board
SAE: Serious adverse event
SBP: Systolic blood pressure
SDM: Substitute Decision Maker
TaARI: Thrombosis and Atherosclerosis Research Institute
TCPS2: Tri-Council Policy Statement: Ethical Conduct for Research Involving Humans
USCOM^TM^: Ultrasonic Cardiac Output Monitor
