# Supplementary material for: A trial to determine whether septic shock-reversal is quicker in pediatric patients randomized to an early goal-directed fluid-sparing strategy versus usual care (SQUEEZE): study protocol for a pilot randomized controlled trial
Source: Trials. 2016 Nov 22;17:556. doi: 10.1186/s13063-016-1689-2 (PMC5120449; doi:10.1186/s13063-016-1689-2)
Supplement: Additional file 2: — Title of data: items from the World Health Organization Trial Registration Data Set. Description of data: items from the World Health Organization Trial Registration Data Set. (PDF 110 kb) [file 13063_2016_1689_MOESM2_ESM.pdf]

Additional File 2: Items from the World Health Organization Trial Registration Data Set

Item	Description
1. Primary registry and trial-identifying number	Primary Registry: ClinicalTrials.gov<br>Identifying Number: NCT01973907
2. Date of registration in primary registry	October 27, 2013.
3. Secondary identifying numbers	HIREB Project #: 13-295
4. Sources of monetary or material support	Monetary Support<br>i) Hamilton Health Sciences New Investigator Fund (Operating Grant - SQUEEZE: $50,000)<br>ii) Hamilton Health Sciences Research Early Career Award 2013, 2014 (Award – Programmatic Support including SQUEEZE: $100,000)<br>iii) Hamilton Health Sciences Research Strategic Initiatives (Operating Grant – includes SQUEEZE-D: $299,453)<br>iv) Canadian Blood Services/Canadian Institutes of Health Research New Investigator Salary Award 2014-2019 (Award – Programmatic Support including SQUEEZE: $300,000)<br>v) Canadian Child Health Clinician Scientist Program Career Enhancement Program Award 2015-2019 (Award – Programmatic Support including SQUEEZE: $25,000)
5. Primary Sponsor	Hamilton Health Sciences
6. Secondary Sponsor	McMaster University
7. Contact for Public Queries	PI: Dr. Melissa Parker<br>Associate Professor of Pediatrics, McMaster University<br>Staff Physician, McMaster Children's Hospital<br>1280 Main St W, Room 3E-20<br>Hamilton, Ontario<br>L8S4K1<br>Email: parkermj@mcmaster.ca<br>Tel: (905) 521-2100 Ext 76651
8. Contact for Scientific Queries	PI: Dr. Melissa Parker<br>Associate Professor of Pediatrics, McMaster University<br>Staff Physician, McMaster Children's Hospital<br>1280 Main St W, Room 3E-20<br>Hamilton, Ontario<br>L8S4K1<br>Email: parkermj@mcmaster.ca<br>Tel: (905) 521-2100 Ext 76651
9. Public title	Pilot study for the SQUEEZE Trial

10. Scientific title	Pilot study for the SQUEEZE Trial: a trial to determine whether septic shock reversal is quicker in pediatric patients randomized to an early goal directed fluid-sparing strategy vs. usual care
11. Countries of recruitment	Canada
12. Health condition(s) or problem(s) studied	Pediatric Septic Shock
13. Intervention(s)	At all points, the caring physician is directed to target ACCM hemodynamic goals using the particular strategy to which the patient is allocated.<br>1. **Usual Care Arm**<br>*Tier 1:* Following randomization, further fluid boluses may be liberally administered to treat persistent signs of shock. The need for and/or timing of initiation of vasoactive medication(s) is at the discretion of the treating physician, but vasoactive support should not be initiated until a minimum of 60 mL/kg (or 3 litres for participants ≥ 50 kg) of isotonic fluid bolus therapy [crystalloid (0.9% Normal Saline or Ringers Lactate) and/or colloid (5% Albumin)] has been administered (Includes fluid boluses received in the 6 hours prior to randomization).<br>*Tier 2:* If vasoactive medication(s) are initiated, the decision to administer further isotonic fluid bolus therapy versus escalating vasoactive medication support to target achievement of recommended ACCM hemodynamic goals is at the discretion of the caring physician. No restrictions regarding volume or number of fluid boluses administered.<br>*Intervention end:* When the patient is free from infusion of vasoactive medication support and shock is reversed.<br><br>2. **Fluid Sparing Arm**<br>*Tier 1:* Vasoactive medication support should be initiated immediately following randomization for children with persistent signs of shock despite receiving a minimum of 40 mL/kg (or 2 litres for participants ≥ 50 kg) of isotonic fluid bolus therapy [crystalloid (0.9% Normal Saline or Ringers Lactate) or colloid (5% Albumin)] in the 6 hours prior to randomization.<br>*Tier 2:* Once vasoactive medication(s) have been initiated, these should be preferentially titrated/escalated to target achievement of recommended ACCM hemodynamic goals. Further fluid bolus therapy should be provided only where

	intravascular hypovolemia is judged to be present in order to maintain adequate (but not excess) intravascular volume. Where further fluid bolus therapy is judged to be indicated, aliquots of 5-10 mL/kg (or 250-500 mL for participants ≥ 50 kg) of isotonic crystalloid or colloid can be given with the lowest acceptable volume preferred and the indication for administration documented.<br>*Intervention end:* When the patient is free from vasoactive medication support and shock is reversed.
14. Key inclusion and exclusion criteria	<u>Inclusion Criteria:</u><br>Inclusion Criteria 1 and 3 must be answered YES to be eligible.<br>1. Age 29 days to <18 years of age<br>* 2a. Persistent signs of shock defined as one or more of the following:<br>i) Vasoactive Medication Dependence (need for vasoactive drug for hemodynamic support)<br>ii) Hypotension (systolic and/or mean blood pressure < 5th percentile for age)<br>iii) Abnormal Perfusion, defined as the presence of 2 or more of the following: abnormal capillary refill (CR < 1 second (flash) or CR ≥ 3 seconds (delayed), tachycardia (HR > 95th percentile for age), decreased level of consciousness, or decreased urine output).<br>*2b. Suspected or confirmed septic shock<br>*2c) Fluid Resuscitation Threshold Met. Patient has received within the previous 6 hours a minimum of:<br>i) 40 mL/kg of isotonic crystalloid (0.9% Normal Saline or Ringer's Lactate), and/or colloid (5% albumin) as IV fluid bolus therapy for participants <50 kg.<br>OR<br>ii) 2 litres of isotonic crystalloid (0.9% Normal Saline or Ringer's Lactate), and/or colloid (5% albumin) as IV fluid bolus therapy for participants ≥50 kg.<br>3. Fluid refractory septic shock as defined by the presence of 2a, 2b, and 2c.<br>*Adapted from the International pediatric sepsis consensus conference: definitions for sepsis and organ dysfunction in pediatrics. [1]<br><br><u>Exclusion Criteria:</u><br>i) Patient admitted to the Neonatal Intensive Care Unit (NICU)<br>ii) Full active resuscitative treatment is not within the goals of care

	iii) Shock secondary to causes other than sepsis (i.e. obvious signs of cardiogenic shock, anaphylactic shock, hemorrhagic shock, spinal shock).<br>iv) Patients requiring resuscitation in the Operating room or Post Anesthetic Care Unit.<br>v) Previous enrolment in this trial, where known by the research team
15. Study type	**Allocation:** Randomized<br>**Blinding:** Investigators, Research Staff, and Healthcare Providers are not blinded to participant assignment<br>**Assignment:** Parallel group, 2 study arms<br>**Purpose:** To determine which of the two resuscitation strategies results in the best outcome for infants and children treated for suspected septic shock.<br>**Phase:** Pilot Trial (for Phase III Trial)<br>**Method of Sequence Generation:** Computer Generated Allocation sequence with no stratification or blocking<br>**Method of Allocation Concealment:** Use of a Third party randomization technique
16. Date of First Enrolment	January 7, 2014
17. Target Sample Size	50 participants
18. Recruitment Status	Enrolling as of January 6, 2014.
19 Primary Outcome(s)	**SQUEEZE**: Feasibility of conducting a full scale trial based on 1) Enrolment rate of at least 2 participants/month (2 participants/site/month if additional sites added) and 2) The ability to initiate study procedures within 1 hour of randomization (descriptive)<br><br>**SQUEEZE-D:** To evaluate the feasibility of describing cfDNA in blood samples obtained for clinical purposes at baseline and 24 hours from children enrolled in the SQUEEZE Pilot Trial.
20. Key Secondary Outcomes(s)<br>Clinical Outcomes	**SQUEEZE:** Suitability of proposed eligibility criteria<br>Ability to collect clinical outcome data of interest<br>Assessment of Process, Resource, Management aspects of study feasibility<br><br>**SQUEEZE-D:** Proportion of SQUEEZE participants for whom specimens are obtained at baseline and 24 hours. Assessment of process, resource, management aspect of study feasibility as pertains to obtaining and testing biological samples.
