# Supplementary material for: A trial to determine whether septic shock-reversal is quicker in pediatric patients randomized to an early goal-directed fluid-sparing strategy versus usual care (SQUEEZE): study protocol for a pilot randomized controlled trial
Source: Trials. 2016 Nov 22;17:556. doi: 10.1186/s13063-016-1689-2 (PMC5120449; doi:10.1186/s13063-016-1689-2)
Supplement: Additional file 3: — Title of data: schedule of enrollment, interventions, and assessments. Description of data: schedule of enrollment, interventions, and assessments. (PDF 155 kb) [file 13063_2016_1689_MOESM3_ESM.pdf]

Additional File 3. Schedule of enrolment, interventions, and assessments

		STUDY PERIOD								
	Enrolment	Allo-cation	Post Allocation							Close -out
Timepoint	-t1	0	t1	t2	t3	t4	t5	t…	tx	
**Enrolment:**										
Eligibility Screen	X									
Informed Consent			—	—	—	—	—	—	→	
Allocation		X								
**Interventions:**										
Usual Care		—	—	—	—	—	—	—	→	
Fluid Sparing		—	—	—	—	—	—	—	→	
**Assessments:**										
Baseline variables	X	X								
Initiation of study procedures			X							
Process Feasibility Outcomes	—	—	—	—	—	—	—	—	—	→
Resource Feasibility Outcomes	—	—	—	—	—	—	—	—	—	→
Feasibility of Study Management Outcomes	—	—	—	—	—	—	—	—	—	→
Shock Reversal Outcome Data			—	—	—	—	—	—	→	
Hemodynamic Outcome Data			—	—	—	—	—	—	→	
USCOM Hemodynamic Data			●—●	X	X	X	X	X	X	
*Fluid adminis-tration, losses, and fluid balance outcome data			—	—	—	—	—	—	—	→
Vasoactive medication outcome data			—	—	—	—	—	—	—	→
Adverse Events Related to Fluid Overload					X		X	X	X	X

Adverse Events Related to Vasoactive Medications					X		X	X	X	X
*Positive cultures Antimicrobials					X		X	X	X	X
Laboratory Results					X		X	X	X	X
Clinical Course and Procedures Data									X	X
Available Plasma sent for cfDNA determination	●—	—●			●—	—●				

-t1: Enrolment
0: Time zero (Allocation)
t1: 1 hr post allocation
t2: 12 hrs post allocation
t3: 24 hrs post allocation
t4: 36 hrs post allocation
t5: 48 hrs post allocation
t… schedule repeats according to t2-t:5 until shock reversal is achieved.
Tx: 24 hrs post determination participant has achieved shock reversal
* Data gathered will include data from the 24 hours immediately prior to enrolment, if available.
