# Supplementary figures and images for: A trial to determine whether septic shock-reversal is quicker in pediatric patients randomized to an early goal-directed fluid-sparing strategy versus usual care (SQUEEZE): study protocol for a pilot randomized controlled trial
Source: Trials. 2016 Nov 22;17:556. doi: 10.1186/s13063-016-1689-2 (PMC5120449; doi:10.1186/s13063-016-1689-2)

# Additional File 4: SQUEEZE Study Algorithm as Illustrated in ACCM Guideline Format

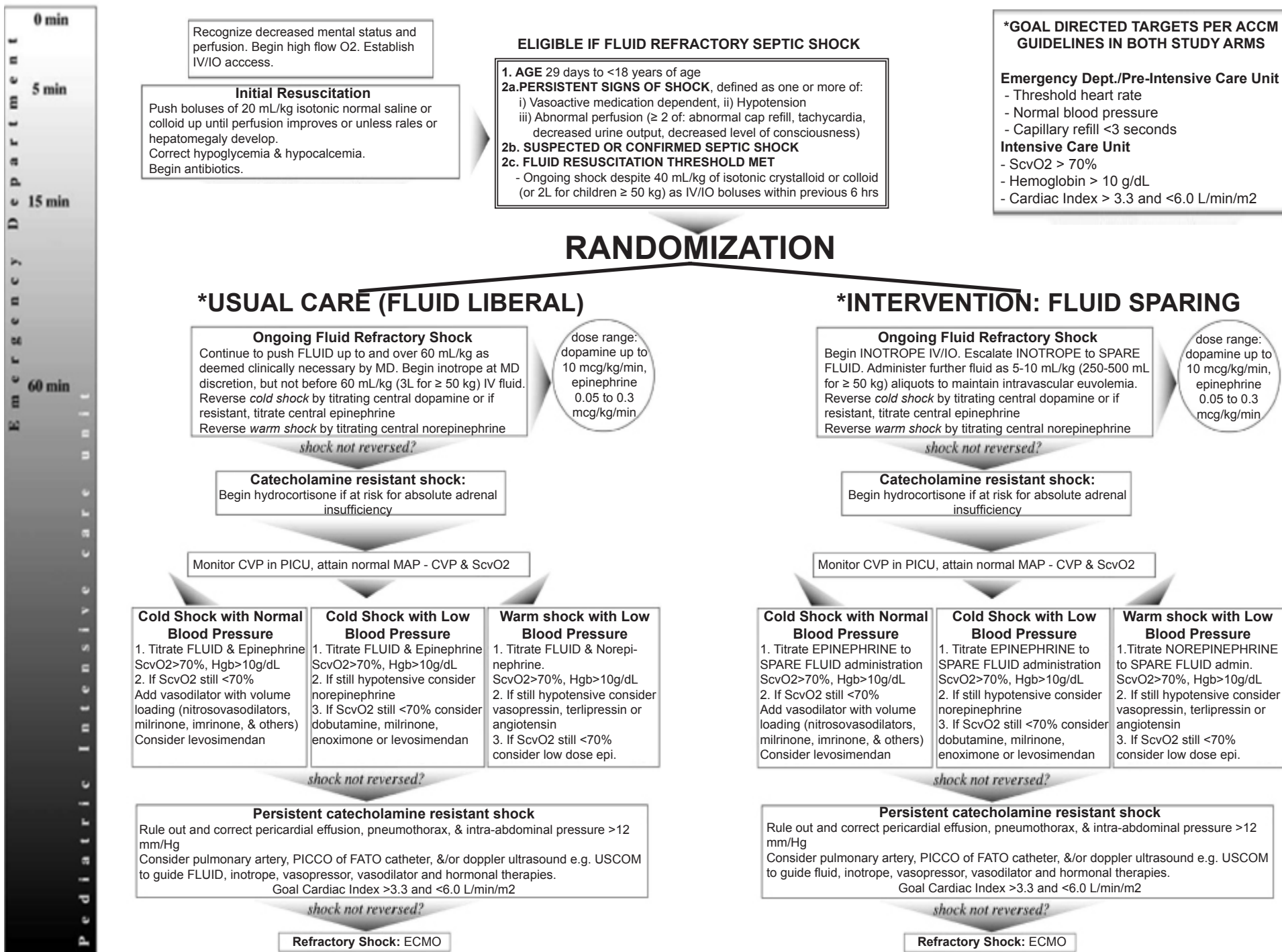

Supplement: Additional file 4: — Title of data: SQUEEZE study algorithm as Illustrated in ACCM Guideline Format. Description of data: SQUEEZE study algorithm as Illustrated in ACCM Guideline Format. (PDF 1162 kb) [file 13063_2016_1689_MOESM4_ESM.pdf]
