# Supplementary material for: A trial to determine whether septic shock-reversal is quicker in pediatric patients randomized to an early goal-directed fluid-sparing strategy versus usual care (SQUEEZE): study protocol for a pilot randomized controlled trial
Source: Trials. 2016 Nov 22;17:556. doi: 10.1186/s13063-016-1689-2 (PMC5120449; doi:10.1186/s13063-016-1689-2)
Supplement: Additional file 5: — Title of data: ACCM Goal Directed Targets from the Surviving Sepsis Guidelines. Description of data: summary of ACCM Goal Directed Targets from the Surviving Sepsis Guidelines [3, 4]. (PDF 78 kb) [file 13063_2016_1689_MOESM5_ESM.pdf]

Additional File 5. ACCM Goal Directed Targets from the Surviving Sepsis Guidelines (3, 4)

Initial Goals (Often, but not exclusively in the Emergency Department setting)
Initial therapies should be directed toward restoring:

- Normal Mental Status
- Threshold Heart Rates
- Peripheral perfusion (capillary refill < 3 seconds)
- Palpable distal pulses
- Normal blood pressure for age.

Age Range	Threshold Heart Rates	*Threshold Perfusion Pressure MAP-CVP (mm Hg)
Term Newborn	120-180	55
Up to 1 year	120-180	60
Up to 2 years	120-180	65
Up to 7 years	100-140	65
Up to 15 years	90-140	65

*Perfusion Pressure = Mean Arterial Pressure (MAP) – Central Venous Pressure (CVP)
**From a practical perspective, perfusion pressure data is frequently not available for children during the initial phase of septic shock resuscitation due to lack of presence of an arterial line and/or central venous catheter. Thus, initial goals of resuscitation may be limited to the other parameters outlined until invasive monitoring is available.*

Subsequent Goals (Intensive Care Unit) Phase
Shock should be further evaluated and resuscitation treatment guided by hemodynamic variables:

- Monitor CVP in PICU (ensure adequate preload)
- Maintain Adequate Perfusion Pressure: Normal MAP-CVP
- Maintain Oxygen Delivery: Mixed Venous Oxygen Saturation [SCVO2] > 70%
- Maintain Oxygen Carrying Capacity: Hemoglobin Concentration > 10 g/dL
